# Effects of Regulatory T Cell Depletion in BALB/c Mice Infected with Low Doses of *Borrelia burgdorferi*

**DOI:** 10.3390/pathogens12020189

**Published:** 2023-01-25

**Authors:** Kaitlyn N. Santiago, Tanya Kozlik, Elizabeth S. Liedhegner, Rebecca A. Slick, Michael W. Lawlor, Dean T. Nardelli

**Affiliations:** 1 Department of Biomedical Sciences, University of Wisconsin–Milwaukee, Milwaukee, WI 53211, USA; 2 Department of Pathology and Laboratory Medicine, Medical College of Wisconsin, Milwaukee, WI 53226, USA; 3 Neuroscience Research Center, Medical College of Wisconsin, Milwaukee, WI 53226, USA; 4 Department of Physiology, Medical College of Wisconsin, Milwaukee, WI 53226, USA; 5 Clinical and Translational Science Institute, Medical College of Wisconsin, Milwaukee, WI 53226, USA

**Keywords:** Lyme disease, Lyme borreliosis, regulatory T cells, Treg cells, arthritis, Lyme arthritis, DEREG, *B. burgdorferi*

## Abstract

We previously demonstrated that a depletion of regulatory T (Treg) cells in Lyme arthritis-resistant C57BL/6 mice leads to pathological changes in the tibiotarsal joints following infection with *Borrelia burgdorferi*. Here, we assessed the effects of Treg cells on the response to *B. burgdorferi* infection in BALB/c mice, which exhibit infection-dose-dependent disease and a different sequence of immune events than C57BL/6 mice. The depletion of Treg cells prior to infection with 1 × 10^2^, but not 5 × 10^3^, organisms led to increased swelling of the tibiotarsal joints. However, Treg cell depletion did not significantly affect the development of histopathology at these low doses of infection. BALB/c mice depleted of Treg cells before infection with 1 × 10^3^ spirochetes harbored a higher borrelial load in the hearts and exhibited higher levels of serum interleukin-10 five weeks later. These results indicate that Treg cells regulate certain aspects of the response to *B. burgdorferi* in a mouse strain that may display a range of disease severities. As the presentation of Lyme disease may vary among humans, it is necessary to consider multiple animal models to obtain a complete picture of the various means by which Treg cells affect the host response to *B. burgdorferi*.

## 1. Introduction

Spirochetes of the *Borrelia burgdorferi sensu lato* complex elicit a series of innate and adaptive inflammatory events that are responsible for the pathology of Lyme disease. Because the symptoms of Lyme disease may vary widely between individuals, differences in the degree to which the host response to borrelial antigens is regulated may be a key determinant of clinical outcomes following infection with Lyme spirochetes. Various regulatory mediators, including the anti-inflammatory cytokine interleukin-10 (IL-10) [1], as well as certain micro RNAs [2], have been shown to inhibit the pathologic response to *B. burgdorferi*. Our early studies demonstrated that an induced subpopulation of CD25-expressing CD4+ cells, which we considered to be putative regulatory T (Treg) cells, also exerts a suppressive effect on the development of experimental disease [3,4,5]. However, these studies were conducted before Foxp3 was characterized as the main phenotypic marker of Treg cells. Although subsequent studies described a correlation between the proportion of Treg cells and a more rapid recovery from antibiotic-refractory Lyme arthritis [6], there had been, until recently, no direct assessment of the role of Treg cells in the context of *B. burgdorferi* infection. 

We showed, using the “depletion of regulatory T cell” (“DEREG”) mouse model, that Treg cells play an important role in preventing the development of pathology in the Lyme arthritis-resistant C57BL/6 mouse strain [7]. DEREG mice express a diphtheria toxin receptor-enhanced green fluorescent protein fusion protein under the control of the Foxp3 promoter; as a result, Treg cells are able to be specifically ablated when these mice are administered low doses of diphtheria toxin (DTx) [8]. Depletion of Treg cells prior to infection of C57BL/6 DEREG mice with *B. burgdorferi* led to significant swelling of the tibiotarsal joints that was maintained for several weeks. In addition, these mice exhibited histopathological changes of the joints in the weeks following infection that did not occur in infected mice with Treg cells intact [7]. These changes occurred after a rebound of the Treg cell population, suggesting that this pre-existing cell population exerts its suppressive effects on the early host response to the spirochete. Moreover, when Treg cells were depleted at various times after infection, a more rapid onset of tibiotarsal joint swelling occurred. These latter findings suggest that, in this arthritis-resistant mouse strain, Treg cells may act as a “guardian” against an accumulating inflammatory potential induced by *B. burgdorferi*, such that their removal results in rapid pathological outcomes.

The availability of DEREG mice on the BALB/c background provides a unique opportunity to further investigate the role of Treg cells in the host response to *B. burgdorferi*. In contrast to C57BL/6 mice and arthritis-susceptible C3H mice, the degree to which arthritis develops in BALB/c mice corresponds to the dose of infection [9]. We show that BALB/c mice depleted of Treg cells and infected with a low dose of *B. burgdorferi* exhibit effects on tibiotarsal joint swelling, spirochetal dissemination, and IL-10 production in ways that are not evident in infected, Treg cell-sufficient mice. However, depletion of Treg cells typically was not sufficient to break BALB/c DEREG mice of their arthritis resistance at the lower infection doses we used. Collectively, our findings suggest that Treg cells may exert their effects differently between mouse strains in the face of *B. burgdorferi* infection, which could be due to genotype-specific differences in proportions and functions of the cells themselves and/or the larger context of attempting to control the genotype-specific host responses to the spirochete. Because Lyme disease does not present uniformly in the population, it is necessary to consider multiple models by which the disease develops and is regulated in order to gain a more complete picture of the complex immune events that may contribute to its pathology.

## 2. Materials and Methods

### 2.1. Mice

Wild-type and DEREG BALB/c (MMRRC stock no. 32049; ref. [8,10]) mice were purchased from The Jackson Laboratory (Bar Harbor, ME). Mice were housed and bred at the University of Wisconsin-Milwaukee Animal Resource Center in a humidity-controlled environment at 21 °C under a 12-h light-and-dark cycle. Food and acidified water were provided ad libitum. Genotypic analysis was used to confirm the presence or absence of the transgene encoding the diphtheria toxin receptor. Male and female mice between 5 and 8 weeks of age were used in these studies. All procedures were approved by the University of Wisconsin-Milwaukee Animal Care and Use Committee.

### 2.2. Organisms and Infection 

B. burgdorferi strain B31-A3 organisms were kindly provided by Dr. Jenifer Coburn (Medical College of Wisconsin). Low-passage (<10) spirochetes were grown in modified Barbour-Stoenner-Kelly (BSK)-H complete medium (Sigma-Aldrich, Inc., St. Louis, MO, USA) at 32 °C until they reached a concentration of 10^6^ microbes/mL before being stored at −80 °C in 1.5 mL screw-top tubes. Aliquots were incubated in BSK medium for approximately 5 days, washed of the media, and, as described previously [11], resuspended in phosphate-buffered saline (PBS) supplemented with heat-inactivated normal mouse serum (NMS) at a concentration of 0.2%. Groups of wild-type and DEREG BALB/c mice were anesthetized with isoflurane and then injected subcutaneously between the scapulae with B. burgdorferi at doses ranging from 1 × 10^2^ to 1 × 10^5^ organisms in a volume of 50 µL of PBS supplemented with NMS. Uninfected mice were injected with PBS supplemented with NMS alone. The motility of B. burgdorferi was confirmed using dark-field microscopy. Organisms were enumerated using a Petroff-Hausser counting chamber.

In a preliminary study, we infected wild-type mice with 1 × 10^3^, 1 × 10^4^, or 1 × 10^5^ *B. burgdorferi* organisms in PBS supplemented with NMS, or with PBS supplemented with NMS alone, to determine the dose of infection to be used in our Treg cell depletion studies. We measured tibiotarsal joint swelling (described below) over the course of 5 weeks as an indication of pathology. Based on this, we used a range of doses of infection (1 × 10^2^–5 × 10^3^ organisms) that would lead to edematous changes of the joint such that any anticipated increases in swelling due to Treg cell depletion would still be apparent. 

### 2.3. Administration of Diphtheria Toxin 

Lyophilized diphtheria toxin (DTx) from *Corynebacterium diphtheriae* (1 mg; EMD Millipore) was resuspended in filter-sterilized PBS and stored at −80 °C. Mice were injected intraperitoneally with 1 µg of DTx in 50 µL PBS, or with PBS alone, for two consecutive days prior to infection with *B. burgdorferi*. Groups consisted of BALB/c DEREG mice administered DTx in PBS with or without subsequent infection, BALB/c DEREG mice administered PBS prior to infection, and wild-type BALB/c mice administered DTx in PBS prior to infection. Mice were weighed immediately before administration of DTx and then regularly thereafter for the duration of the studies. Mice were anesthetized with isoflurane prior to all injections. 

### 2.4. Flow Cytometry

In a preliminary experiment, spleens were collected on the day immediately following the administration of a second dose of DTx in order to assess the efficacy of DTx in depleting Treg cells. A single-cell suspension of splenocytes was prepared by teasing apart the spleens and passing the cells through a 0.2 µm sterile nylon mesh filter into cold Dulbecco Modified Eagle’s Medium (Sigma-Aldrich). Then, 1 × 10^6^ splenocytes were incubated with Fc block (anti-CD16/32), fixed with 2% paraformaldehyde, permeabilized with 0.5% saponin, and stained intracellularly with allophycocyanin (APC)-labeled anti-Foxp3 antibodies (clone FJK-16s; Thermo Fisher Scientific). APC-labeled isotype control antibodies were used to control for nonspecific labeling. Data were acquired as the percentage of Foxp3+ cells among gated lymphocytes using a BD FACSAria (BD Biosciences, San Jose, CA, USA) flow cytometer and FlowJo software (BD Biosciences). 

### 2.5. Assessment of Tibiotarsal Joint Pathology

The width and thickness of the tibiotarsal joints were measured as described previously [3,4,5,12] immediately before, and regularly after, infection. Measurements were made using a digital caliper with a sensitivity of 0.01 mm (Marathon, Richmond Hill, Ontario, Canada) and averaged to provide a mean value for each mouse. Changes in tibiotarsal joint swelling within a mouse were determined by subtracting the baseline value from the value obtained at each time point. The average change in tibiotarsal joint swelling within each group was calculated.

Mice were euthanized via isoflurane inhalation followed by cervical dislocation. One hind paw from each mouse was dissected above the knee joint and placed in cryptically coded tissue embedding cassettes. The paws were fixed in 10% buffered zinc formalin before being embedded in paraffin at the Children’s Hospital of Wisconsin Research Institute’s Histology Core Facility. The embedded tissues were cut into 4-µm sections, placed on glass slides, and stained with hematoxylin and eosin. Blinded histological analysis was performed by a board-certified pathologist (M.W.L.). Histological changes were scored on a 0–3 scale based on the range of observed findings from this study: 0, absence of pathology; 1, a single small focus of inflammation; 2, two or three small foci of inflammation; 3, several larger areas of inflammation.

### 2.6. Determination of Borrelial Load in Tissues 

The remaining hind paw and a bisected portion of the hearts were flash-frozen in liquid nitrogen and then stored at −80 °C to be used for quantitative polymerase chain reaction (qPCR) analysis of bacterial load in these tissues. DNA was extracted using the Qiagen DNeasy Mini Kit (Qiagen, Germantown, MD) according to the manufacturer’s instructions. Extracted DNA was quantified using a NanoDrop One (Thermo Scientific, Waltham, MA, USA). Bacterial load was assessed using a Synergy Brands, Inc. (SYBR) green-based qPCR technique adapted from Morrison, et al. [13]. Primers for the *B. burgdorferi* chromosomal gene *recA* were used [(forward: 5-GCAGCTATCCCACCTTCTTT-3′); (reverse: 5′-ATGAGGCTCTCGGCATTG-3′)]. Values were normalized based on copies of *Mus musculus β-actin* [(forward: 5′-TCACCCACACTGTGCCCATCTACGA-3′); (reverse: 5′-GGATGCCACAGGATTCCATACCCA-3′)]. Standard curves for bacterial and mouse targets were obtained and used as the basis for the quantification of bacterial load within tissue samples. Separate reaction mixtures were prepared for each primer set, with the reaction mixture consisting of 10 µL of PowerUp SYBR Green Master Mix (Life Technologies, Carlsbad, CA), 0.05 µM of both forward and reverse primers for either gene, 1 µL of PCR grade water, 2.5 mM MgCl_2_, and 5 µL of DNA. The amplification protocol consisted of a pre-cycling step at 50 °C for two minutes and an initial denaturation step at 95 °C for two minutes, followed by 40 cycles of denaturation at 95 °C for 15 s and annealing at 60 °C for one minute. A melt curve analysis protocol consisted of 15 s at 95 °C, one minute at 60 °C, then gradually heating to 95 °C for 15 s. Analyses were performed using an Applied Biosystems StepOnePlus^TM^ Real-Time PCR System (Applied Biosystems, Waltham, MA, USA). *B. burgdorferi recA* gene copies were normalized to mouse *β-actin* after being calculated from standard curves.

### 2.7. Assessment of Serum Cytokines 

Blood was collected at the time of euthanasia by intracardiac puncture, and sera were obtained and stored at −80 °C until use. A Mouse Magnetic Luminex Assay Kit (R&D Systems, Minneapolis, MN, USA) was used to determine the concentrations of serum cytokines associated with pro- and anti-inflammatory immune responses. Cytokines examined included granulocyte-macrophage colony-stimulating factor (GM-CSF), interferon-gamma (IFN-γ), IL-1β, IL-2, IL-4, IL-5, IL-6, IL-10, IL-12 p70, IL-13, IL-17A, and tumor necrosis factor alpha (TNF-α). Serum samples were diluted, and a standard curve was created according to the manufacturer’s instructions. A negative control consisting of a calibrator diluent was used to determine the background fluorescence levels. Serum samples were analyzed on a Magpix instrument with xPONENT software (R&D Systems, Minneapolis, MN, USA). Cytokine levels were calculated for each analyte using the respective standard curve. All samples were run in triplicate, and the values were averaged to provide a mean cytokine concentration for each sample.

### 2.8. Assessment of Antibody Levels 

In one study, blood was collected from anesthetized mice via cardiac puncture at the experimental endpoint, 28 days post-infection, and sera were obtained. *B. burgdorferi*-specific total IgG antibody titers were assessed by enzyme-linked immunosorbent assay (ELISA). ELISAs were prepared using sonicated *B. burgdorferi* strain B31-A3 organisms. Whole cell lysates were diluted to a final protein concentration of 0.15625 μg/mL in 0.2M sodium carbonate-bicarbonate buffer. Wells of a 96-well medium-binding polystyrene microtiter plate were coated with antigen and incubated for one hour. Following the coating step, wells were washed and then blocked by adding 300 μL of 2% bovine serum albumin in PBS and incubated at 4 °C overnight. After washing, the following were added sequentially to each well and incubated for one hour at room temperature, with washes between each step: 100 μL of serum serially diluted across the plate, beginning with a 1:200 dilution in the first well; 100 μL of 0.5 mg/mL biotinylated goat anti-mouse IgG Fc diluted 1:50,000 (Southern Biotech, Birmingham, AL, USA), and 100 μL of horseradish peroxidase-conjugated streptavidin diluted 1:2000 (Thermo Fisher Scientific, Waltham, MA, USA). Following this, wells were washed and then incubated with 100 μL 3,3′,5,5′-Tetramethylbenzidine for five minutes and the reaction stopped with 50 μL H_2_SO_4_. The optical density (OD) of each well was read at 450nm. Control wells consisted of no *B. burgdorferi* whole cell lysate; no serum; positive control serum from previously infected mice; negative control serum from uninfected mice; and no IgG Fc antibody. ELISA endpoints were determined by adding three times the standard deviation to the average of the OD readings from all background wells and were reported as the inverse of the last titer to be reported above this value [14]. 

### 2.9. Statistical Analysis

We reasoned, *a priori*, that BALB/c DEREG mice depleted of Treg cells prior to infection with *B. burgdorferi* would exhibit greater differences in the host response than would infected mice not previously depleted of Treg cells. Because both BALB/c DEREG mice administered PBS before infection and wild-type BALB/c mice administered DTx before the infection would have an intact Treg cell compartment, we expected these groups to exhibit similar results in our tests. Therefore, we used the Mann-Whitney U Test (GraphPad Prism) to analyze differences in tibiotarsal joint swelling, histology scores, borrelial load, and cytokine concentrations between Treg cell-depleted, infected mice and each group of Treg cell-sufficient, infected mice. The alpha level was set at 0.05 prior to the initiation of experiments. Data were expressed as mean ± standard error of the mean (SEM) unless otherwise stated.

## 3. Results

### 3.1. Establishment of Infection Dose to Be Used in These Studies

**Figure 1:**
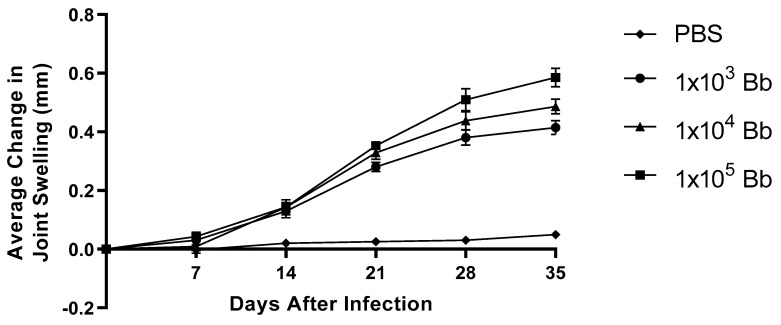
Edematous changes of the tibiotarsal joints following infection with different doses of *B. burgdorferi*. Wild-type BALB/c mice were infected with 1 × 10^5^ (squares, n = 6), 1 × 10^4^ (triangles, n = 6), or 1 × 10^3^ (circles, n = 4) *B. burgdorferi* (Bb) organisms in PBS supplemented with NMS, or injected with PBS supplemented with NMS alone (diamonds, n = 6), to determine the infection doses to be used throughout this study. The width and thickness of the tibiotarsal joints were measured using a digital caliper. Although no statistically significant differences in edema were observed, we chose to use infection doses no greater than on the order of 10^3^ for subsequent experiments. Data are the means of values within each group ± SEM.

It has been established that BALB/c mice possess an innate resistance to developing *B. burgdorferi*-induced arthritis [15] that can be overcome with a large enough inoculum of the spirochete [9]. Therefore, we first established a dose of infection that would yield a moderate degree of tibiotarsal joint pathology, such that any effects of Treg cell depletion would not be obscured by the pathological changes due to the spirochete itself. We infected wild-type BALB/c mice with 1 × 10^3^, 1 × 10^4^, or 1 × 10^5^ *B. burgdorferi* organisms in PBS supplemented with NMS and measured the tibiotarsal joint swelling for five weeks (Figure 1). The development of tibiotarsal joint swelling was evident at each dose two weeks after infection. Following this, swelling increased further in an infection-dose-dependent manner until the end of the experiment; however, the differences in joint swelling between these groups were not statistically significant. Uninfected mice failed to develop edema in the tibiotarsal joints. As a result, we decided to use infection doses spanning (1 × 10^2^ to 5 × 10^3^) the lowest dose we examined here for our Treg cell depletion studies.

### 3.2. Assessment of DTx Function and Effects on Health 

In this study, we used wild-type and DEREG mice on the BALB/c background. DEREG mice express a diphtheria toxin receptor-enhanced green fluorescent protein fusion protein under the control of the Foxp3 promoter. Treg cells are characterized by the expression of Foxp3 [16]; as a result, Treg cells in DEREG mice can be specifically and rapidly ablated upon injection of small doses of DTx [17]. By contrast, wild-type mice do not possess the transgene encoding the DTx receptor. Therefore, in wild-type mice, Treg cells do not express the DTx receptor and, as a result, are unaffected by DTx. In our experiments, we administered 1 μg DTx in PBS, or PBS alone, for two consecutive days before infection of wild-type and DEREG BALB/c mice with *B. burgdorferi*.

We first confirmed our ability to deplete Treg cells with DTx in BALB/c DEREG mice, as we previously have done in C57BL/6 DEREG mice [7]. We used two lots of DTx for our experiments. So, to affirm that the effectiveness of the DTx we used was consistent throughout our work, we determined the proportions of Foxp3+ cells among splenic lymphocytes obtained from BALB/c DEREG mice treated with 1 μg DTx in PBS for two consecutive days or administered PBS alone. After injection of mice with PBS alone, 2.61 ± 0.01% of cells were Foxp3+ one day later. By contrast, one day after administration of DTx in PBS to BALB/c DEREG mice, the proportion of Foxp3+ cells was reduced by approximately 75% (0.66 ± 0.26%) and 80% (0.53 ± 0.18%) with each respective lot of DTx (Figure S1). Therefore, we were able to deplete Treg cells in BALB/c DEREG mice, and our ability to deplete Treg cells was consistent throughout this study. Based on these data, we refer to BALB/c DEREG mice administered DTx as “Treg cell-depleted”.

**Figure 2:**
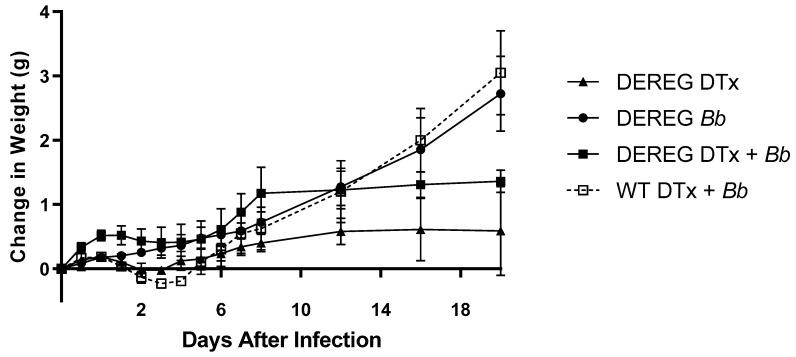
Effects of DTx on weights of mice. DTx in PBS, or PBS alone, were administered to wild-type and DEREG BALB/c mice for two consecutive days prior to infection with 1 × 10^2^ *B. burgdorferi* organisms. The weights of these mice were measured as an indication of overall health upon injection with the toxin. Closed squares, BALBc DEREG mice treated with DTx prior to infection (n = 4); open squares, wild-type BALB/c mice treated with DTx prior to infection (n = 4); circles, BALB/c DEREG mice administered PBS prior to infection (n = 4); triangles, BALB/c DEREG mice treated with DTx without subsequent infection (n = 3). Data are the means of values within each group ± SEM.

DEREG mice treated with 1 µg DTx for two consecutive days are known to exhibit a transient decline in weight or temporary maintenance of weight before gradually recovering [18]. Therefore, we measured the changes in the weights of mice following the injection of DTx as a measure of health in each of our experiments. Our experiments involved the administration of DTx in mice that each was subsequently infected with 1 × 10^2^, 1 × 10^3^, or 5 × 10^3^ *B. burgdorferi* organisms. BALB/c DEREG mice that were administered PBS, without DTx, prior to infection with 1 × 10^2^ *B. burgdorferi* organisms exhibited a consistent weight gain throughout the course of the experiments (Figure 2). By contrast, the weights of DTx-treated BALB/c DEREG mice, with or without subsequent infection, failed to increase for approximately five days. Following this, the weights of each DTx-treated group increased slightly for several days before plateauing for the duration of the experiment. The weights of mice in these groups were less than that of infected DEREG mice that had not previously received DTx. In addition, wild-type mice that received DTx prior to infection initially exhibited a similar pattern of slightly reduced weight for several days. However, the weights of these mice substantially increased over the next few days, at which point their weights were similar to that of infected BALB/c DEREG mice that did not receive DTx. Comparable patterns of weight gain were observed in experiments in which we administered DTx to mice prior to infecting them with 1 × 10^3^ or 5 × 10^3^ *B. burgdorferi* organisms (Figure S2).

### 3.3. Effect of Treg Cell Depletion on Tibiotarsal Joint Swelling 

**Figure 3:**
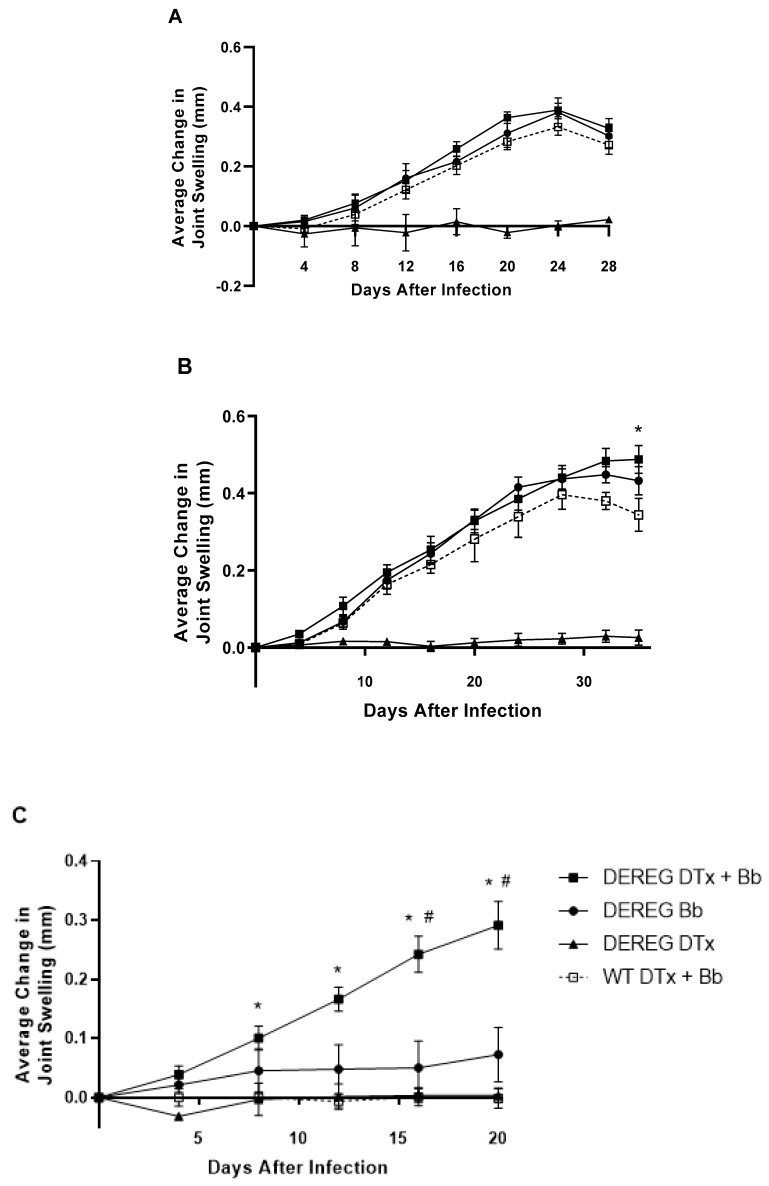
Assessment of tibiotarsal joint swelling. Swelling of the tibiotarsal joints was determined, as described previously, in BALB/c DEREG mice depleted of Treg cells prior to infection with 5 × 10^3^ (**A**), 1 × 10^3^ (**B**), or 1 × 10^2^ (**C**) *B. burgdorferi* organisms. Closed squares, BALBc DEREG mice treated with DTx prior to infection (**A**, n = 8, **B**, n = 7, **C**, n = 4); open squares, wild-type BALB/c mice treated with DTx prior to infection (**A**, n = 8, **B**, n = 4, **C**, n = 4); circles, BALB/c DEREG mice administered PBS prior to infection (**A**, n = 8, **B**, n = 8, **C**, n = 4); triangles, BALB/c DEREG mice administered DTx without subsequent infection (**A**, n = 3, **B**, n = 7, **C**, n = 3). *, significantly greater (*p* ≤ 0.05) swelling in BALB/c DEREG mice treated with DTx prior to infection than in wild-type BALB/c mice treated with DTx prior to infection. #, a statistically insignificant (*p* = 0.057) difference in joint swelling between Treg cell-depleted, infected BALB/c DEREG mice and Treg cell-sufficient, infected BALB/c DEREG mice. Data are the means of values within each group ± SEM. In **A**,**B**, results are the combined data of two independent experiments.

Based on our previous data in Lyme arthritis-resistant C57BL/6 mice [7], we hypothesized that *B. burgdorferi*-infected BALB/c DEREG mice previously depleted of Treg cells would exhibit increases in edematous changes of the tibiotarsal joints. BALB/c DEREG mice infected with 5 × 10^3^ *B. burgdorferi* organisms, without previous Treg cell depletion, exhibited tibiotarsal joint swelling that steadily increased for over three weeks before declining (Figure 3A). A similar course and degree of joint swelling were observed among BALB/c DEREG mice depleted of Treg cells before infection and wild-type BALB/c mice treated with DTx prior to infection. No differences in the level of joint swelling were observed between these groups. DEREG BALB/c mice administered DTx without subsequent infection did not exhibit tibiotarsal joint swelling.

We also determined the effects of Treg cell depletion on BALB/c DEREG mice infected with 1 × 10^3^ *B. burgdorferi* organisms (Figure 3B). Untreated, infected BALB/c DEREG mice exhibited a steady increase in tibiotarsal joint swelling that peaked 32 days after infection before slightly declining. A similar level and pattern of joint swelling were observed in infected wild-type BALB/c mice previously injected with DTx, although their peak of swelling occurred a few days earlier. By contrast, the tibiotarsal joint swelling of BALB/c DEREG mice depleted of Treg cells prior to infection with *B. burgdorferi* increased throughout the five weeks of the experiment. There were no significant differences in the degree of swelling between infected BALB/c DEREG mice that were previously depleted of Treg cells or not. However, we observed a significant (*p* ≤ 0.05) difference between the swelling of DEREG BALB/c mice depleted of Treg cells prior to infection and wild-type BALB/c mice treated with DTx prior to infection. There were no edematous changes of the tibiotarsal joint in uninfected DEREG mice administered DTx. 

We then determined the effect of Treg cells on the development of joint swelling following infection with 1 × 10^2^ *B. burgdorferi* organisms (Figure 3C). Untreated, infected BALB/c DEREG mice developed minimal swelling of the tibiotarsal joints, as did wild-type mice administered DTx prior to infection. By contrast, BALB/c DEREG mice depleted of Treg cells prior to infection with 1 × 10^2^ spirochetes developed a steady increase in joint swelling that persisted for the 20 days of the study. The increased joint swelling of these Treg cell-depleted, infected DEREG mice 16 and 20 days after infection did not achieve statistical significance (*p* = 0.057) compared to Treg cell-sufficient, infected DEREG mice, but it was significantly greater than that of infected wild-type mice previously administered diphtheria toxin (*p* ≤ 0.05). DEREG mice administered DTx without subsequent infection exhibited no edema in the tibiotarsal joints.

### 3.4. Effect of Treg Cell Depletion on Histopathology of the Tibiotarsal Joints 

**Figure 4:**
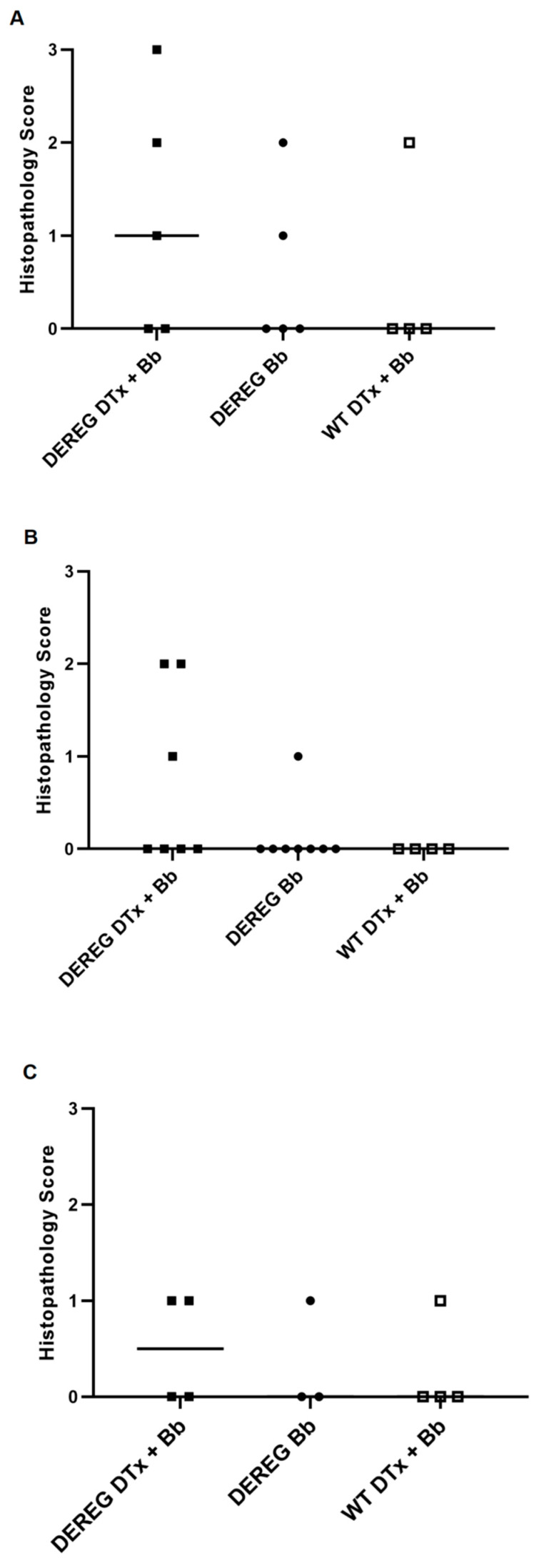
Histopathological scores of the tibiotarsal joints. BALB/c DEREG and wild-type mice were administered DTx in PBS or PBS alone prior to infection with 5 × 10^3^ (**A**), 1 × 10^3^ (**B**), or 1 × 10^2^ (**C**) *B. burgdorferi* organisms. Histological changes were scored on a 0–3 scale based on the range of observed findings from this study: 0, absence of pathology; 1, a single small focus of inflammation; 2, two or three small foci of inflammation; 3, several larger areas of inflammation. “DEREG DTx + Bb”, DEREG BALB/c mice depleted of Treg cells prior to infection; “DEREG Bb”, DEREG BALB/c mice administered PBS prior to infection; “WT DTx + Bb”, wild-type BALB/c mice treated with DTx prior to infection. Bars indicate the median score. In **B**, the results are the combined data of two independent experiments. The medians of some groups are 0; as a result, these bars overlap with the x-axis.

We did not observe a significant effect of Treg cell depletion on the development of tibiotarsal joint histopathology at any infection dose of *B. burgdorferi* we used. In fact, many mice developed no histopathological changes and, in those that did, their arthritis was generally mild. However, at least some degree of histopathology was observed in 60% (3 of 5) of BALB/c DEREG mice depleted of Treg cells and infected with 5 × 10^3^ *B. burgdorferi* organisms (Figure 4A). One mouse in this group exhibited moderate histopathology, as reflected by several larger areas of inflammation in the tissue. The other two mice in this group with histopathological changes of the joint developed a single focus or just a few foci of inflammation. Mild arthritis developed in 40% (2 of 5) of infected, Treg cell-sufficient BALB/c DEREG mice and in 25% (1 of 4) of wild-type BALB/c mice treated with DTx prior to infection.

Similarly, we observed mild histopathological changes of the tibiotarsal joints in 43% (3 of 7) of Treg cell-depleted, BALB/c DEREG mice infected with 1 × 10^3^ *B. burgdorferi* organisms (Figure 4B). By contrast, 12.5% (1 of 8) of infected, T cell-sufficient BALB/c DEREG mice exhibited mild arthritis. However, these differences were not statistically significant. Wild-type BALB/c mice administered DTx prior to infection with 1 × 10^3^ organisms did not develop any degree of histopathology. In addition, only mild arthritis was found in 50% (2 of 4) of Treg cell-depleted BALB/c DEREG mice infected with 1 × 10^2^ spirochetes, 33% (1 of 3) of Treg cell-sufficient, infected DEREG mice, and 25% (1 of 4) of wild-type mice treated with DTx prior to infection (Figure 4C). 

### 3.5. Spirochete Load in Tissues

**Figure 5:**
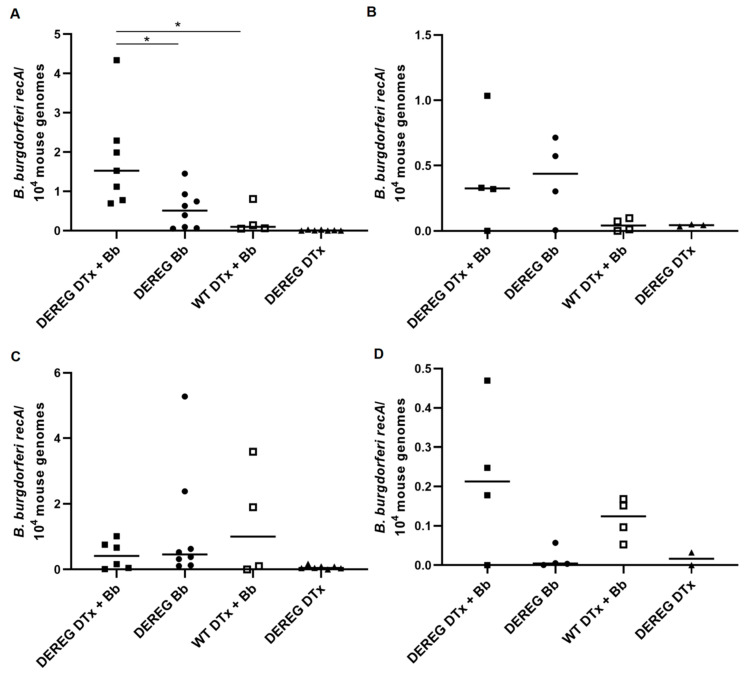
Quantification of borrelial load in tissues of mice depleted of T cells prior to infection with *B. burgdorferi*. Levels of the *B. burgdorferi* gene *recA* in the hearts and tibiotarsal joints of mice were normalized to *Mus musculus β-actin*. Data were acquired from the hearts (**A**,**B**) and tibiotarsal joints (**C**,**D**) of mice infected with 1 × 10^3^ *B. burgdorferi* organisms and euthanized 35 days later (**A,C**) or of mice infected with 1 × 10^2^ *B. burgdorferi* organisms and euthanized 20 days later (**B**,**D**). “DEREG DTx + Bb”, DEREG BALB/c mice depleted of Treg cells prior to infection (**A**,**C**, n = 7; **B**,**D**, n = 4); “DEREG Bb”, DEREG BALB/c mice administered PBS prior to infection (**A**,**C**, n = 8; **B**,**D**, n = 4); “WT DTx + Bb”, wild-type BALB/c mice treated with DTx prior to infection (n = 4); “DEREG DTx”, DEREG BALB/c mice depleted of Treg cells without subsequent infection (**A**,**C**, n = 7; **B**,**D**, n = 3). *, *p* ≤ 0.05. Bars represent the median of each group. In **A**,**C**, the results are the combined data of two independent experiments. Not shown in **C** is one mouse in the “DEREG DTx + Bb” group in which exceptionally high levels of *recA* (406/10^4^ mouse genomes) were detected. In **D**, one joint from the “DEREG DTx” group was unable to be located among our samples and, as a result, was not measured. The heart tissue from this mouse is shown in **B**. Our analysis did not indicate significant differences between comparison groups in **D**.

We determined the level of borrelial load in the tibiotarsal joints and hearts of mice 35 days after infection with 10^3^ organisms or 20 days after infection with 1 × 10^2^ organisms. As expected, the presence of spirochetes in these tissues was low, overall, but greater in mice infected with the higher dose of *B. burgdorferi*. There were significantly more (*p* ≤ 0.05) *B. burgdorferi* organisms in the hearts of BALB/c DEREG mice depleted of Treg cells prior to infection with 1 × 10^3^ *B. burgdorferi* organisms than in untreated, infected BALB/c DEREG mice and in wild-type mice treated with DTx prior to infection (Figure 5A). By contrast, there were no significant differences in borrelial load in the joints of mice infected with 1 × 10^3^ *B. burgdorferi* organisms (Figure 5C); however, there were indications that, in some mice, possession of Treg cells favored migration of spirochetes to the tibiotarsal joint. Additionally, no significant differences in the microbial load were observed in either the hearts (Figure 5B) or joints (Figure 5D) of mice infected with 1 × 10^2^ *B. burgdorferi* organisms. However, there were indications that, in some mice, depletion of Treg cells favored migration of spirochetes to the tibiotarsal joint. No organisms were detected in uninfected BALB/c mice administered DTx alone.

### 3.6. Cytokine Levels 

**Figure 6:**
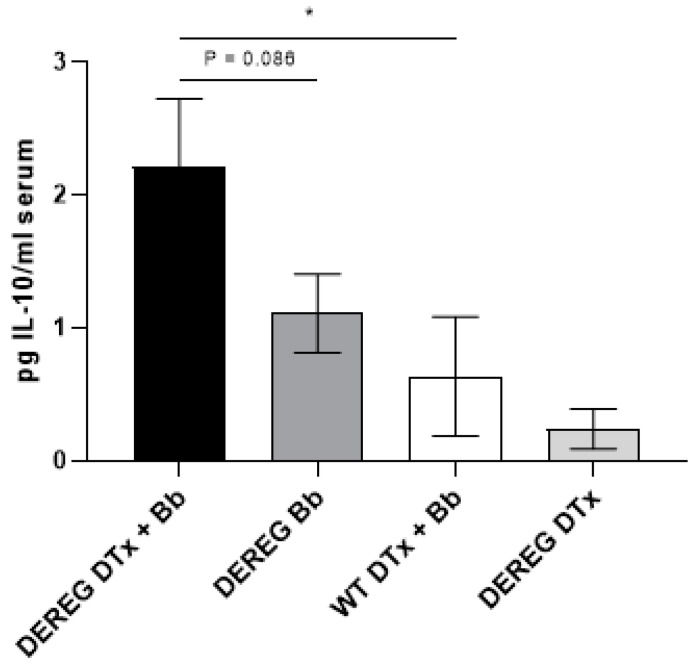
Concentrations of serum IL-10 in mice depleted of Treg cells prior to infection with *B. burgdorferi*. DTx in PBS or PBS alone was administered to mice prior to infection with 1 × 10^3^ *B. burgdorferi* organisms in PBS supplemented with NMS. Sera were obtained 35 days after infection. “DEREG DTx + Bb”, DEREG BALB/c mice depleted of Treg cells prior to infection (n = 7); “DEREG Bb”, DEREG BALB/c mice administered PBS prior to infection (n = 8); “WT DTx + Bb”, wild-type BALB/c mice treated with DTx prior to infection (n = 4); “DEREG DTx”, DEREG BALB/c mice depleted of Treg cells without subsequent infection (n = 6). Data are shown as mean values within each group ± SEM. *, *p* ≤ 0.05. The results are the combined data of two independent experiments. An insufficient amount of serum was obtained in one mouse in the “DEREG DTx” group.

We used a multiplex assay to determine the concentrations of several cytokines in the serum of Treg cell-depleted and Treg cell-sufficient mice that were infected with 1 × 10^3^ *B. burgdorferi* organisms and euthanized 35 days later. The majority of the cytokines we analyzed were not detected in the serum; however, IL-10 was present in low amounts. A significantly (*p* ≤ 0.05) higher concentration of IL-10 was observed in Treg cell-depleted, *B. burgdorferi*-infected BALB/c DEREG mice than in wild-type mice treated with DTx prior to infection (Figure 6). In addition, a greater concentration of IL-10 was detected in Treg cell-depleted, infected DEREG mice than in their T cell-sufficient counterparts; however, this difference was not statistically significant (*p* = 0.086).

### 3.7. Antibody Titers 

**Figure 7:**
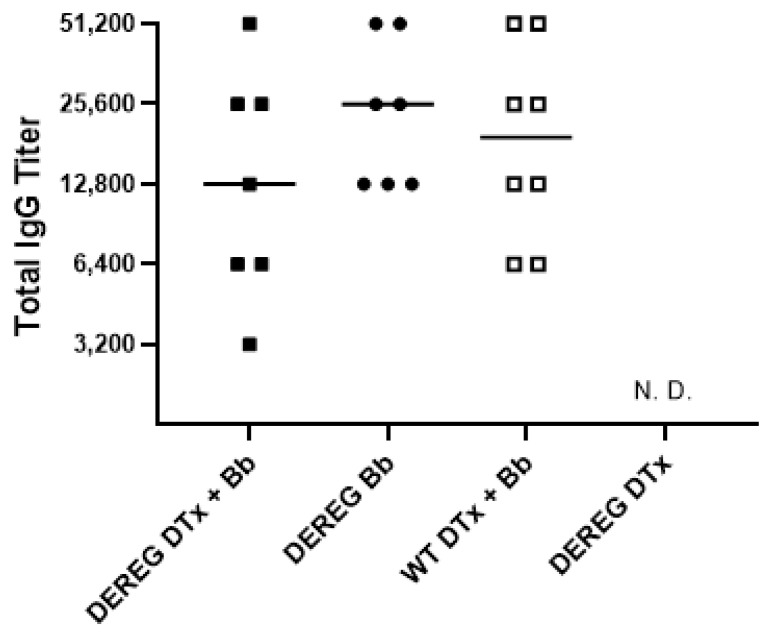
Total *B. burgdorferi* IgG titers in mice depleted of Treg cells prior to infection with *B. burgdorferi*. Sera were obtained 28 days after infection of Treg cell-depleted or Treg cell-sufficient BALB/c mice with 5 × 10^3^ *B. burgdorferi* organisms. No significant differences in IgG titers were observed between groups. “DEREG DTx + Bb”, DEREG BALB/c mice depleted of Treg cells prior to infection; “DEREG Bb”, DEREG BALB/c mice administered PBS prior to infection; “WT DTx + Bb”, wild-type BALB/c mice treated with DTx prior to infection; “DEREG DTx”, DEREG BALB/c mice depleted of Treg cells without subsequent infection. N. D., not detected. The results are the combined data of two independent experiments.

We determined the titers of total *B. burgdorferi*-specific IgG antibodies 28 days after infection with 5 × 10^3^ organisms (Figure 7). We observed no statistically significant differences in antibody levels between groups, regardless of whether the infected BALB/c mice were depleted of Treg cells or not. However, the titers of Treg cell-sufficient, infected BALB/c DEREG mice tended to be concentrated at a higher level than those of the other groups. No *B. burgdorferi*-specific IgG antibodies were detected in uninfected BALB/c DEREG mice depleted of Treg cells.

## 4. Discussion

Several host factors contribute to pathological outcomes of *B. burgdorferi* infection in humans. As Treg cells are capable of controlling both innate and adaptive immune responses to pathogens, it is plausible that Treg cells are one such factor that influences whether, and the degree to which, an infected patient develops symptoms of Lyme disease. Indeed, it has been shown that differences in the number [6] and functional capacity [19] of Treg cells in patients with antibiotic-resistant Lyme arthritis are associated with differences in their clinical outcomes post-treatment. Our recent findings [7] showed that even a transient depletion of Treg cells prior to, or following, infection of Lyme arthritis-resistant C57BL/6 mice leads to a pathology of the tibiotarsal joints. This confirmed our early studies [3,4,5] and implicated Treg cells as a key host factor in the pathological host response to *B. burgdorferi*.

Here, we determined the effects of Treg cells on the response following infection with low (1 × 10^2^–5 × 10^3^) doses of spirochetes using BALB/c mice. These mice reflect a different predisposition to disease and a different sequence of immune events than C57BL/6 mice. Early studies demonstrated that BALB/c mice develop relatively mild arthritis following intraperitoneal inoculation with high numbers (1 × 10^6^–1 × 10^7^) of an infectious *B. burgdorferi* isolate [15]. It was subsequently shown, via intradermal infection with pathogenic strains of the spirochete, that this resistance occurs at a lower (2 × 10^2^) inoculum; this resistance can be overcome as the dose of infection increases [9]. We found that Treg cell depletion prior to infection of BALB/c mice allowed for the development of edema in the joints at a very low (1 × 10^2^) infection dose of *B. burgdorferi.* In addition, Treg cell-depleted BALB/c DEREG mice infected with 1 × 10^3^ spirochetes maintained their swelling for at least 35 days, unlike our groups of infected, Treg cell-sufficient mice. However, we observed no significant differences in the development of joint swelling when Treg cells were depleted from DEREG mice infected with 5 × 10^3^ organisms. It is possible that, just as there is a critical infection dose that is sufficient to overcome the resistance to tibiotarsal joint swelling and pathology in BALB/c mice, there may be a critical level of relative Treg cell presence that is required to maintain that resistance. Moreover, our results also suggest that the immune events stimulated by increasing antigenic challenge are able to overcome this suppressive effect of Treg cells. However, in contrast to our findings in C57BL/6 mice, Treg cells of BALB/c mice did not appear to contribute greatly to resistance in histopathological changes of the tibiotarsal joints. We observed no statistically significant differences in the severity of histopathology between groups of infected mice that were Treg cell-sufficient or previously depleted of Treg cells. However, we observed that a higher percentage of Treg cell-depleted mice infected with 1 × 10^3^ spirochetes developed at least some pathology than did infected Treg cell-sufficient mice. Overall, however, many of our mice failed to develop any degree of arthritis, and, of those that did, the disease was mild. We did observe moderate disease in a single mouse that was depleted of Treg cells prior to infection with 5 × 10^3^ *B. burgdorferi* organisms. A wide range of arthritic severity also was previously reported in BALB/cJ mice infected with 2 × 10^3^ *B. burgdorferi* N40 organisms [20], illustrating certain challenges with using the BALB/c mouse in this system. In our studies using C57BL/6 mice, we found that depletion of Treg cells in the days prior to infection resulted in significantly greater joint swelling, as well as histological changes of the joints, two weeks later [7]. Collectively, these findings suggest that Treg cells may function differently between mouse strains in the face of *B. burgdorferi* infection, which could be due to genotype-specific differences in proportions and functions of the cells themselves [21] and/or the larger context of attempting to control the genotype-specific immune responses to the spirochete. Regardless of the explanation, our current and previous findings provide support for the assertion that Treg cells may be one of several variables of the host that affect the response to *B. burgdorferi,* and that the extent of these effects may depend at least in part on the genotype of the host.

One factor to account for in the current study is the Th2 bias exhibited by BALB/c mice [22], which frequently opposes Th1 responses. Th1 responses are associated with Lyme arthritis in humans and in arthritis-susceptible C3H mice [23,24,25,26]. It has been shown that the resistance to the development of Lyme arthritis in C57BL/6 mice can be attributed significantly to a greater anti-inflammatory IL-10 response than that observed in C3H mice [1]. Specifically, IL-10 has been shown to be a significant regulator of IFN-γ production among cells of patients with Lyme arthritis and in the context of experimental Lyme arthritis [27,28,29]. In addition, it has been shown that human Th1 cells are more amenable to the inhibitory effects of Treg cells than Th2 cells [30]. We showed that depletion of Treg cells prior to infection of C57BL/6 mice not only increased pathology in the tibiotarsal joints but also created an environment in which restimulated cells produced more IFN-γ and less IL-10 [7]. Therefore, the resistance to Lyme arthritis in C57BL/6 mice may be attributed at least in part to the degree to which IL-10 controls a pre-existing potential to launch pathological Th1 responses. By contrast, BALB/c mice are inclined to develop a Th2 response over a Th1 response, including in response to *B. burgdorferi* [26]. Administration of IL-4-neutralizing antibodies in vivo increased the tibiotarsal joint swelling and borrelial load [26], demonstrating a more protective role for Th2 responses, and, specifically, IL-4, in these mice. This mechanism of opposing Th1 responses is different from that observed in C57BL/6 mice. In depleting Treg cells (and reducing the amount of IL-10 they produce) in BALB/c mice, it is possible that a stronger Th2 response ensued, such that no differences in histopathology would be evident in the weeks following *B. burgdorferi* infection. Moreover, Th2 cells also produce IL-10, and the production of Th2 cell-derived IL-10 could potentially be enhanced as Treg cells that may be controlling them are removed. Additionally, we previously observed that the histopathology in C57BL/6 mice depleted of Treg cells prior to infection eventually resolved by about 4 weeks after infection [7]. This suggests that the rebounding Treg cells in these mice were functional. In the current study, we observed higher levels of serum IL-10 after five weeks in mice that had been depleted of Treg cells prior to infection with 1 × 10^3^ *B. burgdorferi* organisms than in infected, Treg cell-sufficient mice. It is possible that the higher concentration of serum IL-10 in these mice was the result of a Treg cell rebound that was compensatory in nature and which supplemented the IL-10 that was already existing due to the inherent Th2 bias of these mice.

It is interesting to ponder the effect that Treg cells may have on affecting the dissemination of the spirochete to different tissues. We generally detected more *B. burgdorferi* in the tibiotarsal joints of Treg cell-depleted mice subsequently infected with 1 × 10^2^ organisms than in infected mice with an intact Treg cell compartment. However, this difference was not statistically significant, likely due to the absence of spirochetes in a single Treg cell-depleted mouse. Treg cell depletion also did not have any significant effect on the ability of *B. burgdorferi* to disseminate to the heart following this lower dose of infection; however, some individual Treg cell-sufficient mice harbored more organisms in this tissue. While depletion of Treg cells in mice infected with 1 × 10^3^ spirochetes also did not affect dissemination to the tibiotarsal joint, it did lead to a significantly greater presence of *B. burgdorferi* in the heart. The borrelial loads in all of these mice were low, which, given our relatively low doses of infection, perhaps is not surprising. We detected higher levels of *B. burgdorferi* in the tissues of mice that were infected with the larger inoculum, which has been observed previously [9]. However, *B. burgdorferi* does not disseminate in BALB/c mice as well as they do in C3H mice, at least following infection with doses on the order of 10^2^–10^3^ organisms [9,20]. Our findings suggest that, in some cases, the activity of pre-existing Treg cells in BALB/c mice may actually provide some advantage to the host in reducing the dissemination of the spirochete to the tissues. This may be supported by the levels of total *B. burgdorferi*-specific IgG we detected, in which infected, Treg cell-sufficient mice tended to exhibit higher titers than Treg cell-depleted mice. However, these differences in antibody titers were not statistically significant. Future studies examining the effect of Treg cells in BALB/c mice infected with higher doses of *B. burgdorferi* would provide additional information on this system, particularly in the context of presumably heightened inflammation and pathology.

In conclusion, we show that at a low dose of *B. burgdorferi* infection in BALB/c mice, Treg cells function to prevent the development of edema in the tibiotarsal joints. Depletion of Treg cells had minor, but not significant, effects on the development of histopathology in the joints and facilitated the migration of spirochetes from the site of infection to the heart. However, the depletion of Treg cells prior to infection did not significantly impact the development of total IgG production against *B. burgdorferi* but did result in an eventual increase in the levels of serum IL-10. Collectively, these results indicate that Treg cells regulate certain aspects of joint pathology and microbial dissemination in the context of a murine model that can display a range of disease severities. These findings complement our previous work which showed, using a Lyme arthritis-resistant mouse strain, that Treg cells contribute to the protection against arthritis. Future studies will determine whether our findings are due to inherent, genotype-specific differences in Treg cell number and function, or if the broader immunological milieu determines the degree to which Treg cells are able to function. As the presentation of Lyme disease varies in the human population, it is necessary to consider multiple animal models of the disease to obtain a more complete picture of the various means by which Treg cells participate in the response to *B. burgdorferi*.

## Data Availability

The data presented in this study are available upon request from the corresponding author.

## Supplementary Materials

The following are available online at https://www.mdpi.com/article/10.3390/pathogens12020189/s1, Figure S1: Depletion of Foxp3+ cells using DTx, Figure S2: Effects of DTx on weights of mice.
