# Supplementary material for: Effects of Regulatory T Cell Depletion in BALB/c Mice Infected with Low Doses of Borrelia burgdorferi
Source: Pathogens. 2023 Jan 25;12(2):189. doi: 10.3390/pathogens12020189 (PMC9965304; doi:10.3390/pathogens12020189)
Supplement: Supplementary file 1 [file pathogens-12-00189-s001.zip › pathogens-2116709-supplementary.pdf]

Supplementary Data, Santiago and Kozlik, *et al.*

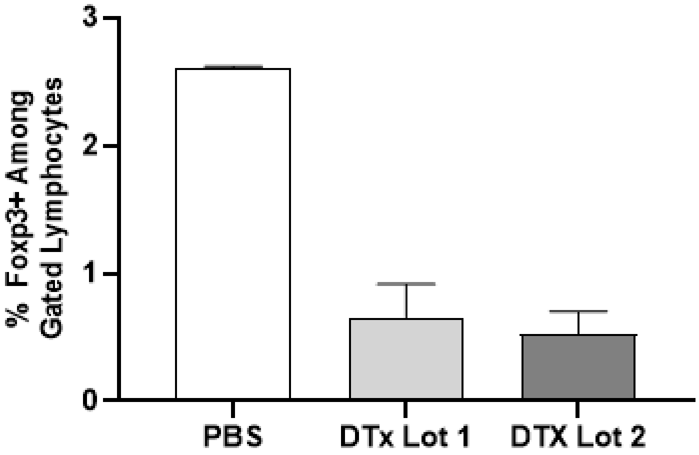

Figure S1. Depletion of Foxp3+ cells using DTx. Two lots of DTx were used in this study. Proportions of Foxp3+ cells among splenic lymphocytes obtained from BALB/c DEREG mice administered 1μg DTx in PBS for two consecutive days or administered PBS alone are shown. After injection of mice with PBS alone, 2.61% ± 0.01% of cells were Foxp3+ one day later (n = 2). By contrast, one day after administration of DTx in PBS to BALB/c DEREG mice, the proportion of Foxp3+ cells was reduced by approximately 75% (0.66% ± 0.26%; n = 3) and 80% (0.53% ± 0.18%; n = 3) with each respective lot of DTx.

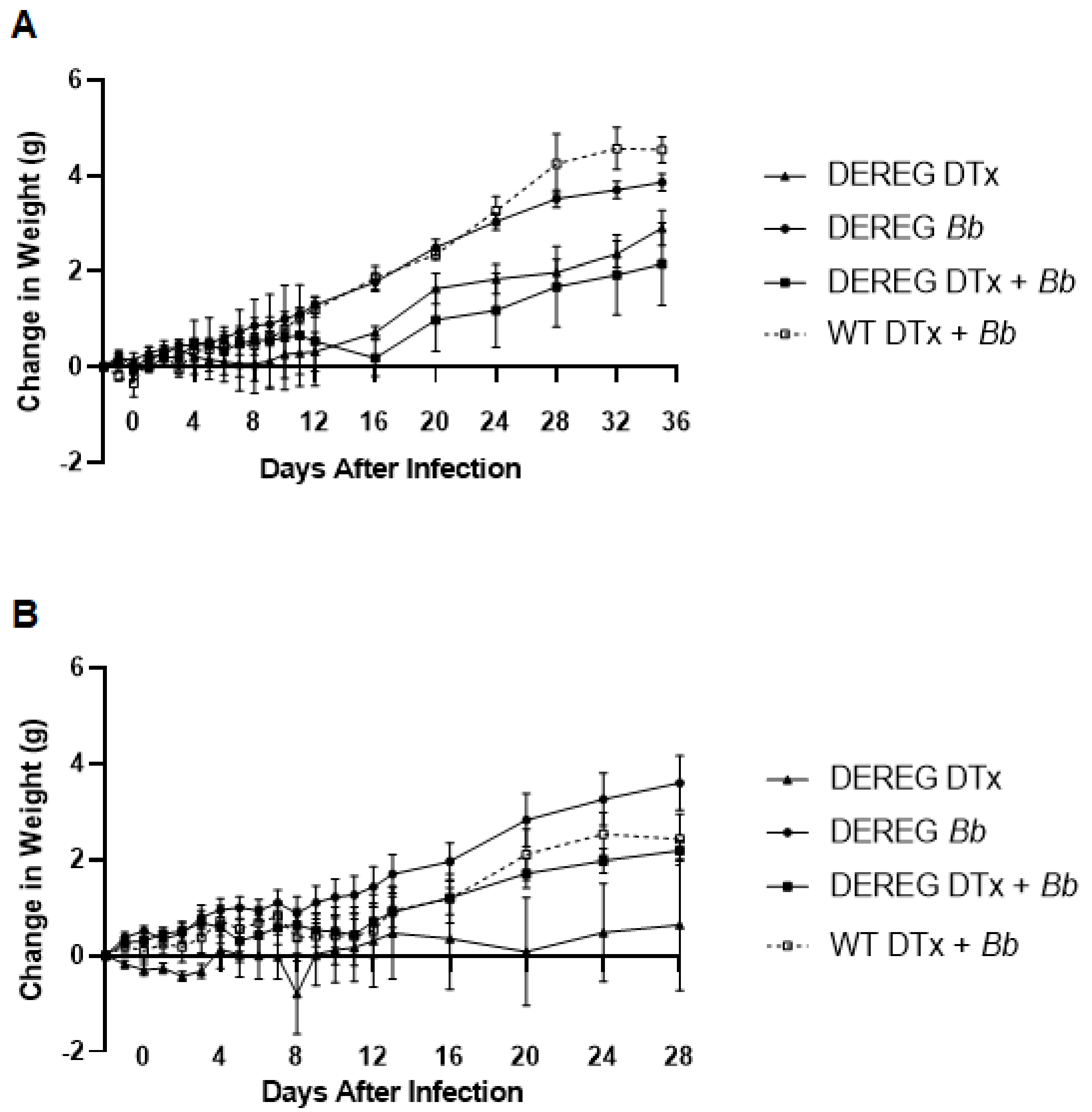

Figure S2. Effects of DTx on weights of mice. DTx in PBS, or PBS alone, were administered to wild-type and DEREG BALB/c mice for two consecutive days prior to infection with 1×10^3 (A) or 5×10^3 (B) *B. burgdorferi* organisms. Weights of these mice were measured as an indication of overall health upon injection with the toxin. Closed squares, BALBc DEREG mice treated with DTx prior to infection (A, n = 7; B, n = 8); open squares, wild-type BALB/c mice treated with DTx prior to infection (A, n = 4; B, n = 8); circles, BALB/c DEREG mice administered PBS prior to infection (A, n = 8; B, n = 8); triangles, BALB/c DEREG mice administered DTx

without subsequent infection (A, n = 7; B, n = 3). Data are the means of values within each group ± SEM.
